# Respiratory particle emission rates from children during speaking

**DOI:** 10.1038/s41598-023-45615-0

**Published:** 2023-10-25

**Authors:** Elisa Caracci, Luca Stabile, Andrea R. Ferro, Lidia Morawska, Giorgio Buonanno

**Affiliations:** 1 Department of Civil and Mechanical Engineering, University of Cassino and Southern Lazio, Cassino, FR Italy; 2 Department of Civil and Environmental Engineering, Clarkson University, Potsdam, NY USA; 3 International Laboratory for Air Quality and Health, Queensland University of Technology, Brisbane, QLD Australia

**Keywords:** Environmental sciences, Health care, Engineering

## Abstract

The number of respiratory particles emitted during different respiratory activities is one of the main parameters affecting the airborne transmission of respiratory pathogens. Information on respiratory particle emission rates is mostly available for adults (few studies have investigated adolescents and children) and generally involves a limited number of subjects. In the present paper we attempted to reduce this knowledge gap by conducting an extensive experimental campaign to measure the emission of respiratory particles of more than 400 children aged 6 to 12 years while they pronounced a phonetically balanced word list at two different voice intensity levels (“speaking” and “loudly speaking”). Respiratory particle concentrations, particle distributions, and exhaled air flow rates were measured to estimate the respiratory particle emission rate. Sound pressure levels were also simultaneously measured. We found out that median respiratory particle emission rates for speaking and loudly speaking were 26 particles s^−1^ (range 7.1–93 particles s^−1^) and 41 particles s^−1^ (range 10–146 particles s^−1^), respectively. Children sex was significant for emission rates, with higher emission rates for males during both speaking and loudly speaking. No effect of age on the emission rates was identified. Concerning particle size distributions, for both respiratory activities, a main mode at approximately 0.6 µm and a second minor mode at < 2 µm were observed, and no differences were found between males and females. This information provides important input parameters in predictive models adopted to estimate the transmission risk of airborne pathogens in indoor spaces.

## Introduction

The SARS-CoV-2 pandemic highlighted the importance of the airborne transmission route for respiratory pathogens^1–6^. Airborne transmission is caused by the emission of virus-laden respiratory particles by infected subjects during their respiratory activities (e.g., breathing, speaking, singing, coughing, sneezing). Recent studies have highlighted that, for a given exposure scenario (e.g., volume of the indoor space, distance amongst subjects, ventilation of the indoor space, exposure time, etc.), the risk of infection of exposed subjects is mostly affected by the strength of the emission^7–13^ and, thus, by the number of respiratory particles emitted during a specific respiratory activity. Consequently, the pertinence of predictive models (e.g., zero-dimensional well-mixed models or three-dimensional close-contact models) in estimating the risk of infection for specific exposure scenarios strongly relies upon the knowledge of the emission of respiratory particles^7,8,14,15^. Several papers have described the formation (in the respiratory tract) and the consequent emission of respiratory particles during different respiratory activities^16–27^. These studies show that different physical processes occurring in different regions of the respiratory system lead to the formation of specific particle size modes. The three main modes that have been identified are those involving particles forming in the bronchioles, larynx, and mouth^16,17^. Nonetheless, the measurement of respiratory particle emissions is not an easy task because the particles undergo sudden thermodynamic processes (e.g., complex and interconnected effects of inertia, gravity, and evaporation) leading to possible artefacts when measured in ambient air after the emission^28–30^.

Studies that focus on the evaluation of respiratory particle emissions can be classified in two main categories: fluid-dynamic visualization and respiratory particle quantification. Fluid-dynamic visualizations (e.g., high-speed photography, particle image velocimetry etc.) provide useful information for understanding the dynamics of the exhaled flow as soon as it is released in ambient air, and also show how far the particles can travel^3,16,21^. However, they do not provide quantification of the particle emissions. In contrast, respiratory particle quantification studies are conducted using particle counters (e.g., with different measurement techniques, including optical and time-of-flight methods) to measure respiratory particle number concentrations and size distributions in the exhaled air^17,22,23,30–32^. Nonetheless, these challenges in measuring the respiratory particles and the difficulty of recruiting and working with human participants have resulted in a limited number of studies reporting on respiratory particle emission characteristics with a limited number of subjects (generally fewer than 20) and a range of different methodologies. The experimental apparatus has varied; in some studies volunteers were asked to speak into a sampling funnel directly connected to the particle counter (the experiments were conducted in a HEPA filtered laminar flow hood, in a cleanroom, or in ambient air)^17,24,25,33^. In other studies the subjects had to speak into a large duct, where a constant airflow was generated by suction of a filter fan unit, and the measurement probe was placed at a certain distance from the volunteer^22,23^. Other researchers adopted a small wind tunnel equipped with HEPA filters into which the volunteers placed their head to perform respiratory activities while the particle counter sampled the aerosol from the wind tunnel^31,32^. There have also been major differences in the testing protocols. For example, respiratory activities (breathing, speaking, singing, coughing, and shouting) were performed by pronouncing specific phonemes^25,26^ or multiphonetic text (e.g., “Rainbow passage”)^26^ with or without sound pressure level control^27^. Finally, volunteers involved in the studies have mainly included adolescents^22^ and adults^26,32,34^, whereas little information on children aged 12 years and younger is currently available^17,23,35^.

In the present study, more than 400 children attending primary and secondary schools (aged 6 to 12) were involved in an experimental study aimed at providing emission rates of respiratory particles while speaking at two different intensity levels—“speaking” and “loudly speaking”. To this purpose experimental apparatus and testing protocol were optimized and, indeed, respiratory particle emission rates were obtained by directly measuring respiratory particle concentration and exhaled flow rate while subjects pronounced a phonetically balanced word list.

## Materials and methods

To provide respiratory particle emission rates (i.e., respiratory particles emitted per unit time), two different tests were performed: (a) measurement of the air flow velocity exhaled by children while speaking and loudly speaking (to estimate the exhaled air flow rate) and (b) measurement of the respiratory particle concentration while speaking and loudly speaking. Tests were performed on more than 400 children aged 6 to 12 years attending primary and secondary schools in Cassino (FR), Central Italy. After the collected data were carefully examined, 371 measurements/children were considered valid for data post-processing. In particular, we excluded from the analysis the data/children for whom instrument issues (e.g., missing data not recorded by the instruments) and/or test issues (children performing one of the tests improperly) occurred. Details of the investigated population of children who provided valid measurements are provided in Table 1.

**Table 1:** Details (sex and age) of the investigated population (only children whose data were considered valid).

Entire population		371
Sex	Male	49.9%
	Female	50.1%
Age (years)	6	11.1%
	7	11.3%
	8	14.8%
	9	10.2%
	10	9.4%
	11	27.5%
	12	15.6%

Air velocity and particle concentration measurement tests were performed while children read the phonetically balanced word list typically adopted for word recognition testing in Italian. The word list is as follows: “*papà, babbo, tetto, dado, cocco, lago, ciccio, Gigi, mamma, nonna, fifa, viva, sasso, rosa, sciocco, zia, zanzara, Lulù, ramarro, rana, giugno, luglio, strada, spruzzo, completo, taxi, cosmopolita, sardanapalo, Nabucodonosor, Afghanistan*”. The word list was distributed in the days before the test so that the children could train in reading it with the support of their teachers. This was extremely important for younger children (e.g. 6 years old students) as they were not skilled enough to read properly.

### Human subjects

The ethical committee of the University of Cassino and Southern Lazio approved this study (Report no. 4 of 2023 emitted by ethical committee), indeed, the research was performed in accordance with relevant guidelines and regulations of the committee. Informed consent from the parents of the recruited children was obtained prior to study participation.

### Evaluation of the exhaled air flow rate

**Figure 1:**
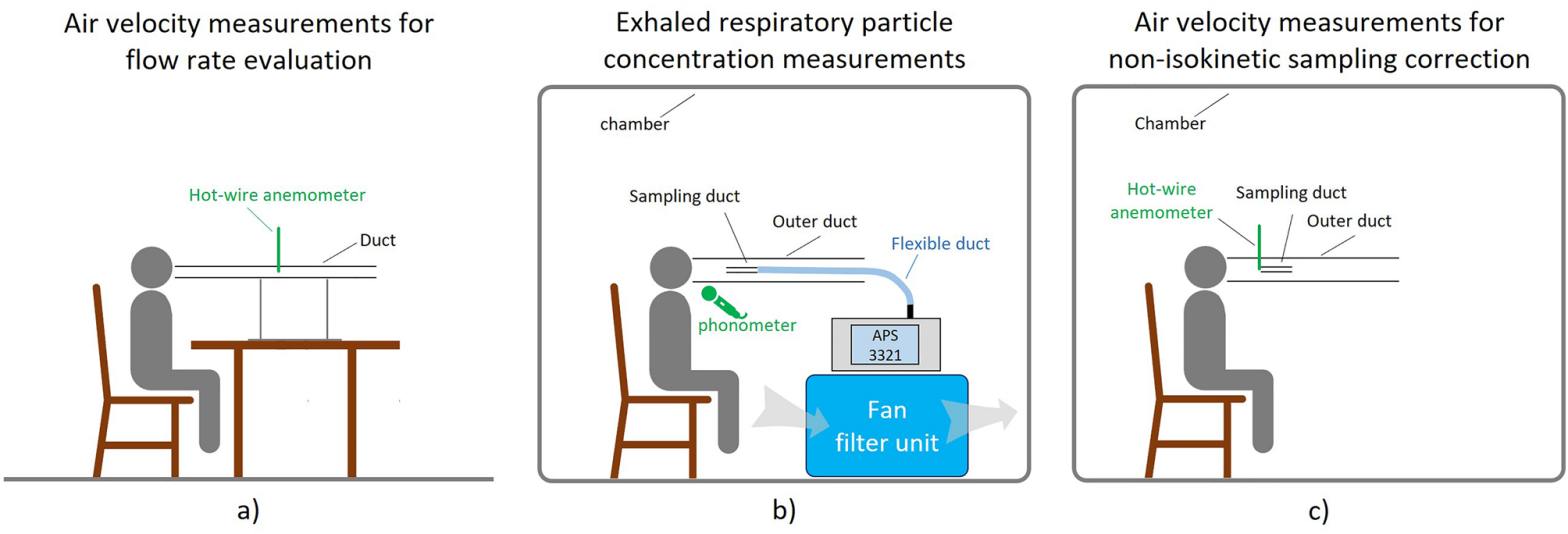
Experimental set up adopted to measure (**a**) air velocity to estimate the air flow rates, (**b**) respiratory particle concentrations and, (**c**) air velocity for non-isokinetic sampling correction while speaking and loudly speaking.

The velocity of the exhaled air while speaking at two different intensities (test a) was measured with a Testo 405i Smart Probe hot-wire anemometer (measurement range 0–30 m s^−1^; resolution 0.01 m s^−1^) recently calibrated by the manufacturer. The children were asked to read the word list twice consecutively at their normal intensity level (referred to as “speaking”) and twice at a higher intensity level (referred to as “loudly speaking”). The children were not asked to speak at a specific sound pressure level but just to read the list at their own speaking and loudly speaking sound pressure level. During the test they had to speak as close as possible to the inlet of a duct (diameter 0.0465 m; length 1 m) as illustrated in Fig. 1a; the air velocity sampling point was placed at the center of the section (i.e., maximum velocity) and at a distance of 40 cm from the inlet to guarantee fully developed flow in the duct. Air velocity measurements were performed with a 1-s sampling frequency. The air flow rates for speaking and loudly speaking were evaluated by multiplying the average velocity at the center of the duct during the test by the cross-sectional area of the duct and by the average-to-maximum velocity correction factor (which is 0.5 because the flow through the duct was laminar)^36^.

### Measurement of the respiratory particle concentration

The respiratory particle concentration exhaled by children while speaking (test b) was measured by an aerodynamic particle sizer spectrometer (APS 3321, TSI Inc.) which measures particle number distribution and total concentration in the 0.5–20 µm diameter range, with a sampling flow rate of 5 L min^−1^, on the basis of a time-of-flight technique. The APS was recently calibrated by the manufacturer to guarantee the quality of the data. To avoid miscounting due to the presence of environmental airborne particles, the test was conducted in a 1.80 m × 1.20 m × 2.20 m plexiglass chamber with a fan filter unit (FFU) to reduce the background particle concentration level. The FFU is a 55 cm × 74 cm × 60 cm parallel pipe equipped with a HEPA H14 filter plus an F7 pre-filtration stage and characterized by an adjustable flow rate (up to 850 m^3^ h^−1^). To guarantee accurate particle sampling, the apparatus shown in Fig. 1b was set up. In particular, children were asked to speak as close as possible to a 15-cm diameter duct (the outer duct). A further sampling duct was placed inside the outer duct at 10 cm from the outer duct inlet section and connected to the APS through a 40-cm flexible duct. The sampling duct was printed by a 3D printer using a biodegradable thermoplastic aliphatic polyester. The 4-cm diameter duct was designed to carry out isokinetic sampling; indeed, the air velocity resulting from the APS flow rate (about 0.07 m s^−1^) was the average air velocity typically produced by children while speaking as determined from preliminary tests previously performed on 10 children.

The following procedure was adopted for the test: (i) the FFU was run at the maximum flow rate for 3 min to reduce the airborne particle background concentration in the chamber; (ii) the FFU was switched off and a 30-s background concentration measurement (with a 1-s sampling frequency) was carried out; (iii) particle concentrations and distributions were measured with a 1-s sampling frequency while the child being tested read the word list twice consecutively at his/her normal intensity level (speaking); (iv) the FFU was run at the maximum flow rate for 3 min to reduce the airborne particle background concentration in the chamber; (v) the FFU was switched off and a 30-s background concentration measurement (with a 1-s sampling frequency) was carried out; (vi) particle concentrations and distributions were measured with a 1-s sampling frequency while the child read the word list twice at a higher intensity level (loudly speaking). The children were asked to speak and loudly speak with the same sound pressure level as during test a. The median duration of each air velocity (test a) and particle concentration/distribution measurement (test b), i.e. reading the word list twice consecutively, was 56 s (with corresponding 5th–95th percentile range of 44–143 s). Nonetheless, the length of the measurement is not expected affecting the results as both, air velocity and particle concentrations/distributions, are measured with a 1-s sampling frequency.

The voice intensity level during the tests was also measured with a Delta Ohm Class 1 HD2110 (Geass; declared uncertainty < 0.7 dB) adopting a A-weighted sound pressure level and post-processing the data as 1/3 octave band. The phonometer was placed 10 cm from the child’s mouth. Average intensity levels while speaking and loudly speaking were obtained to normalize to the average background intensity level inside the chamber; indeed, the phonometer was switched on during the entire procedure (steps i-to-vi).

The respiratory particle emission rates for speaking and loudly speaking were evaluated by multiplying the estimated air flow rates by the respiratory particle concentrations. The estimated air flow rates were corrected for possible non-isokinetic sampling as described below on the basis of actual air velocity measurements. The respiratory particle concentrations while speaking (step iii of the measurement procedure) and loudly speaking (step vi of the measurement procedure) were provided as average values and were normalized to the corresponding background concentration measured during steps ii and v of the measurement procedure, respectively. In other words the average value of the background concentration measured during steps ii and v were subtracted from the values measured during steps iii and vi. The resulting average particle concentrations were corrected for particle losses as described below.

### Corrections for non-isokinetic sampling and particle losses

As mentioned above, isokinetic sampling is a critical aspect to be considered when sampling air flow in ducts. This is why we designed a sampling duct with a cross-sectional area that could guarantee nominal isokinetic sampling. Nonetheless, the air velocity while speaking can vary significantly amongst children, so corrections for non-isokinetic sampling need to be considered. This is why we also measured the air velocity while speaking at the inlet section of the sampling duct for each child, and for both speaking and loudly speaking while using the hot-wire anemometer as depicted in Fig. 1c. In particular, air velocity measurements were performed while the child being tested read the word list twice consecutively. The average velocities resulting from the tests were adopted to perform the isokinetic correction. Thus, the correction factor of the particle concentration due to possible non-isokinetic sampling was evaluated according to the formulation proposed by Xu^37^, which is a function of the difference between the actual speaking velocity and the sampling velocity and the Stokes number. Every measured concentration was corrected on the basis of the measured air velocity for both speaking and loudly speaking; corrections applied ranged between 0.8 and 4.0%.

Sampling and particle losses during transport through the inlet system were considered and corrected through a recently developed particle loss calculator multifunctional software tool^38^. The tool takes into account the different particle loss mechanisms (diffusion, sedimentation, turbulent inertial deposition, inertial deposition due to bend and contraction) occurring in the entire sampling line. The corrections calculated (and applied) were all < 1%.

### Statistical analysis

All the data (sound pressure level, air velocity, respiratory particle concentrations, emission rates) were tested through a preliminary normality test (Shapiro–Wilk test with a 95% confidence level; i.e., *p*-value < 0.05) to check if they followed a Gaussian distribution. Sound pressure level data followed a Gaussian distribution; therefore statistical analyses were carried out using analysis of variance (ANOVA) and a post-hoc Tukey–Kramer test^39^ with a 95% confidence level (i.e., *p*-value < 0.05). Other parameters that did not follow a Gaussian distribution were analyzed using a non-parametric test and a further post-hoc test (Kruskal–Wallis test)^40^**,** adopting a 95% confidence level (i.e., *p*-value < 0.05).

## Results and discussion

### Air flow rate and sound pressure level

The exhaled air flow rates and sound pressure levels produced by children while speaking and loudly speaking are presented in Table 2 for females and males. The median exhaled air flow rate values for speaking activities were 0.28 and 0.31 m^3^ h^−1^ for females and males, respectively, and were not significantly different. Similarly, there were no significant differences between females and males in the exhaled air flow rate values for loudly speaking (median values of 0.31 and 0.34 m^3^ h^−1^). Median exhaled air flow rates were also the same for speaking and loudly speaking for females and males. In contrast, a slight age effect was recognized: older children (11 and 12 years old) recorded statistically higher flow rates than younger children (6 years old) as reported in Table 2.

**Table 2:** Median (and 5th–95th percentile) values of sound pressure level, exhaled flow rate, particle concentration, and particle emission rate while speaking and loudly speaking for the entire investigated population and as a function of sex and age.

Population		Sound pressure level (dB)		Flow rate (m^3^ h^−1^)		Particle concentration (particles cm^−3^)		Emission rate (particles s^−1^)	
		Speaking	Loudly speaking	Speaking	Loudly speaking	Speaking	Loudly speaking	Speaking	Loudly speaking
Entire population		80.0 (73.1–87.4)*	86.3 (79.5–95.1)*	0.31 (0.15–0.55)	0.34 (0.15–0.60)	0.30 (0.11–0.94)*	0.43 (0.15–1.37)*	26 (7.1–93)*	41 (10–146)*
Sex	Female	79.4 (72.5–86.3)*^+^	85.5 (79.4–92.8)*^·^	0.28 (0.15–0.49)	0.31 (0.15–0.55)	0.29 (0.10–0.72)^Δ^	0.37 (0.15–1.14)^+^	23 (7.0–62)*^+^	33 (8.9–98)*^·^
	Male	80.9 (74.3–88.3)^Δ+^	88.1 (79.6–95.8)^Δ·^	0.31 (0.15–0.61)	0.34 (0.15–0.61)	0.33 (0.11–1.09)*^Δ^	0.53 (0.18–1.40)*^+^	28 (7.7–105)^Δ+^	51 (13–162)^Δ·^
Age	6	78.9 (72.4–87.3)	84.6 (77.0–91.0)	0.21 (0.12–0.40)*^Δ^	0.21 (0.12–0.44)*^Δ^	0.32 (0.16–1.92)	0.56 (0.24–2.53)*^Δ^	24 (7.1–165)	43 (11–164)
	7	81.6 (76.4–87.3)	86.6 (81.3–95.4)	0.24 (0.12–0.43)^+^	0.31 (0.15–0.49)	0.37 (0.12–0.90)	0.61 (0.20–1.58)	23 (8.8–106)	48 (12–148)
	8	81.1 (73.4–89.0)	87.4 (81.4–94.1)	0.31 (0.17–0.53)	0.34 (0.18–0.53)	0.37 (0.14–0.83)*	0.60 (0.18–1.41)^Δ^	30 (12–82)	54 (14–129)
	9	79.6 (75.1–88.6)	86.3 (81.0–94.8)	0.31 (0.18–0.50)	0.31 (0.18–0.51)	0.26 (0.11–0.92)	0.40 (0.18–1.02)	22 (6.7–77)	33 (12–126)
	10	79.2 (72.9–88.2)	84.8 (78.7–95.4)	0.28 (0.17–0.36)	0.34 (0.15–0.47)	0.27 (0.11–0.69)	0.53 (0.13–1.35)	22 (5.8–71)	41 (7.3–148)
	11	79.7 (73.2–86.0)	86.8 (79.9–96.0)	0.34 (0.15–0.67)^Δ+^	0.37 (0.18–0.70)*	0.29 (0.10–0.73)	0.39 (0.15–1.37)	28 (7.5–86)	41 (11–154)
	12	80.0 (74.0–86.3)	85.8 (79.1–94.6)	0.32 (0.15–0.59)*	0.39 (0.18–0.62)^Δ^	0.24 (0.10–0.68)*	0.32 (0.16–0.97)*	20 (7.7–81)	31 (13–112)

Symbols (*, Δ, + , ·) indicate data pairs with significant differences.

Sound pressure levels were significantly different between females and males for both speaking (median values 79.4 and 80.9 dB, respectively) and loudly speaking (85.5 and 88.1 dB, respectively). Moreover, sound pressure levels were statistically different (for both females and males) between speaking and loudly speaking. No age effect was detected as the sound pressure levels were the same for children in all age groups (for speaking and loudly speaking separately).

### Respiratory particle concentrations and size distributions

Respiratory particle concentrations (normalized with respect to the background concentrations) as a function of sex and speaking activity are shown in Table 2. Values for females and males were significantly different for loudly speaking (median values 0.37 and 0.53 particles cm^−3^, respectively) but not for speaking (median values 0.29 and 0.33 particles cm^−3^, respectively). The respiratory particle concentrations were statistically different (for both females and males) between speaking and loudly speaking (please see the “entire population” data in Table 2). Such differences as a function of the speaking activity were also recognized in previous studies^17,31–33^. However, an accurate comparison of the measured concentrations between our study and previous studies cannot be easily achieved because of the different methodologies applied in the experimental studies in terms of experimental apparatus, respiratory activity, and type of volunteers. Nonetheless, a rough comparison with previous studies reveals that the particle concentrations we measured for children were within the ranges measured for similar speaking activities performed by adolescents and adults^24,25,27,31–33^ as shown in Table 3.

**Table 3:** Comparison of respiratory particle concentrations and/or emission rates with previous studies (for speaking activities only).

Author	Respiratory activity	Participants	Particle concentration or emission rate
This study	Speaking, i.e., reading a text at normal and higher intensity level	371 children	Concentrations: ∙ Speaking = 0.11–0.94 particles cm^−3^ (median 0.30 particles cm^−3^) ∙ Loudly speaking = 0.15–1.37 particles cm^−3^ (0.43 particles cm^−3^) Emission rates: ∙ Speaking = 7.1–92.5 particles s^−1^ (median 25.6 particles s^−1^) ∙ Loudly speaking = 10.4–146.2 particles s^−1^ (median 40.6 particles s^−1^)
Mürbe et al.^22^	Speaking, i.e., reading a text	8 adolescents	Emission rate: 16–267 particles s^−1^ (median 80 particles s^−1^)
Ahmed et al.^25^	Phonation (specific frequencies and different vocal intensity levels)	40 adults	Emission rate: 2.1–22 particles s^−1^ (median 9.1 particles s^−1^)
Bagheri et al.^17^	Speaking, i.e., reading a text at normal and higher intensity level	132 subjects including 21 children aged 5–9 years old and 24 children aged 10–14 years	Concentrations: ∙ Speaking 5–9 years = geometric mean ± deviation 0.041 ± 1.654 particles cm^−3^ ∙ Speaking 10–14 years = geometric mean ± deviation 0.077 ± 2.324 particles cm^−3^ ∙ Loudly speaking 5–9 years = geometric mean ± deviation 0.072 ± 1.859 particles cm^−3^ ∙ Loudly speaking 10–14 years = geometric mean ± deviation 0.120 ± 2.187 particles cm^−3^ Concentration increases with age (e.g., adults)
Asadi et al.^33^	Speaking, i.e., reading a text	48 adults	Concentrations: 0.06–3 particles cm^−3^ Emission rates: 1–50 particles s^−1^
Archer et al.^27^	Speaking at a controlled sound pressure level	Adolescents aged 12–14 years and adults aged19-72 years	Concentrations: ∙ Adolescents = 0.10–0.36 particles cm^−3^ (median 0.21 particles cm^−3^) ∙ Adults = 0.02–1.70 particles cm^−3^ (median 0.18 particles cm^−3^) Emission rates: ∙ Adolescents = 19.7–74.5 particles s^−1^ (median 40.7 particles s^−1^) ∙ Adults = 11.4–306 particles s^−1^ (median 60.1 particles s^−1^)
Fleischer et al.^23^	Speaking, i.e., reading a text	15 children aged 8–10 years	Emission rates: 8–86 particles s^−1^ (median 24 particles s^−1^)
Morawska et al.^32^	Speaking, i.e., voiced counting	15 adults	Concentrations: median 0.3 particles cm^−3^
Gregson et al.^24^	Speaking at different volumes	25 adults	Concentrations: 0.016–3.7 particles cm^−3^ (median, at 70–80 dB, 0.22 particles cm^−3^)

Our results also showed a slight age effect on the respiratory particle concentrations. Indeed, 12-year-old children had significantly lower concentrations than 8-year-old children for speaking, and also 6- and 8-year-old children for loudly speaking (Table 2). This is a novel finding that should be explored in future research because the few previous studies involving children^17,23^ reported a respiratory particle concentration increase as a function of age, although this was not adequately justified. However, these studies involved a small number of volunteers that limited statistical comparisons. Tavares et al. (2012) reported that the efficiency of the respiratory mechanism during phonation in children aged > 10 years was different from that of children aged < 10 years^41^. In addition, children aged 6–8 years typically lose their central incisors^42^, reducing the physical barrier for respiratory particle emission. Nonetheless, the larynx and pulmonary system grow and mature through puberty, and this may also affect aerosol formation^43,44^. Thus, further studies should be carried out to evaluate if these or other phenomena actually affect respiratory particle emission, also taking note of possible phonetic difficulties of the children (this was not considered in the present paper).

**Figure 2:**
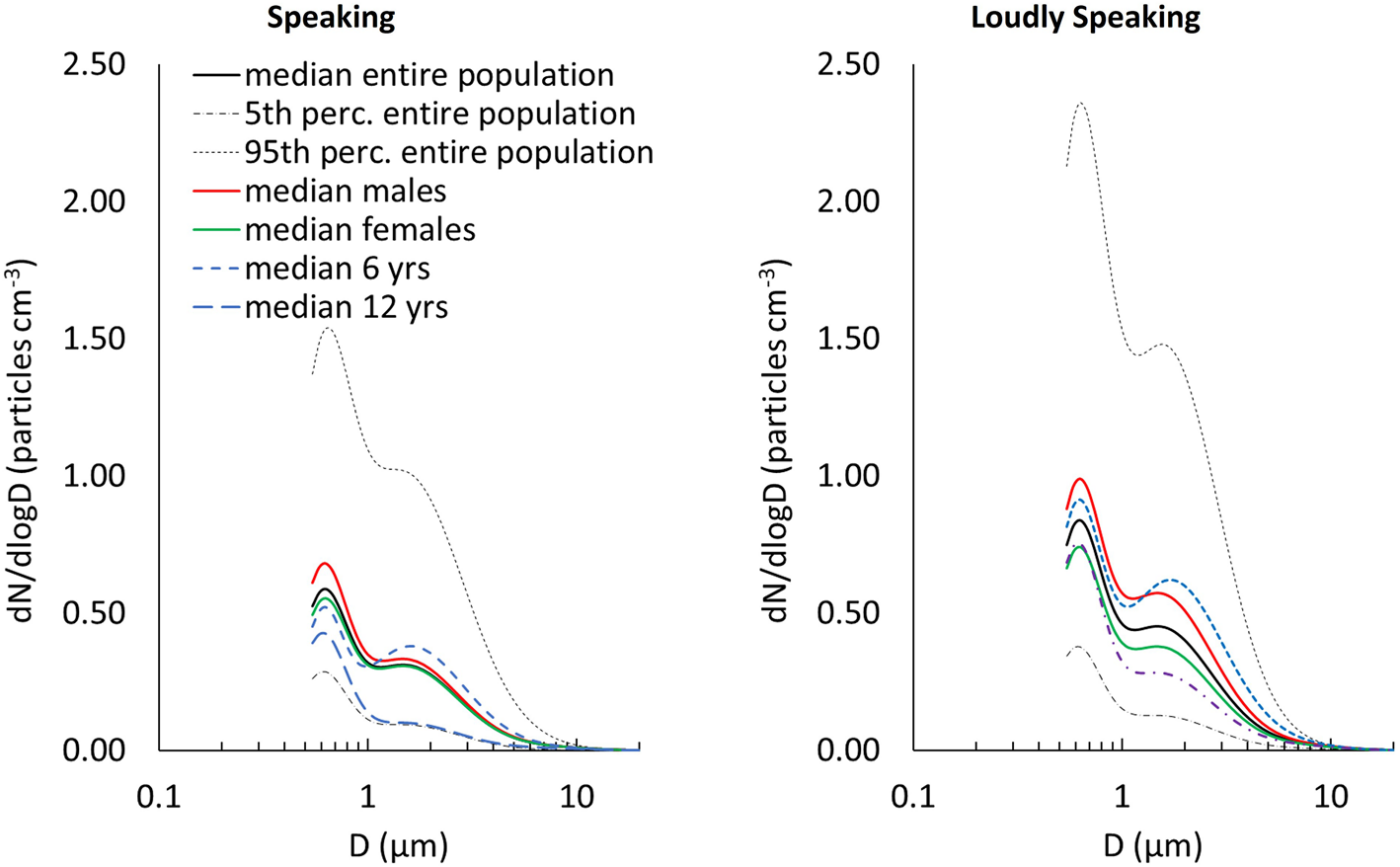
Median respiratory particle size distributions for speaking (left) and loudly speaking (right) activities for the entire population, males, females, 6-year-old children, and 12-year-old children. 5th and 95th percentile distributions for the entire population are also shown.

Figure 2 shows the median (and corresponding 5th–95th percentile range) respiratory particle size distributions measured for the entire population investigated for speaking and loudly speaking activities. Both distributions present a main mode at approximately 0.6 µm and a second minor mode at < 2 µm. Similar distributions were obtained in previous studies, independent of frequency and loudness^17,22,25,33^. In particular, the main mode is characteristic of the respiratory particles generated in the bronchioles (and it is present also in breathing activities), whereas the second mode is generally associated with the generation occurring in the larynx and pharynx which are more typical of speaking and singing activities^17^. In Fig. 2, median distributions for females and males are also reported. These distributions were similar for the activities of boys and girls; thus, sex was not a factor affecting particle generation. In contrast, a slight age effect was observed in younger children, in whom the second mode (at < 2 µm) was more pronounced than in older children. This is clearly detectable in Fig. 2 where, for example, median distributions for 6-year-old and 12-year-old children (those characterized by statistically different concentrations) are also reported. This aspect is worthy of further investigation as it was not previously discovered in the few papers reporting particle size distributions for children. In fact, an increase of the second mode was previously shown only in adults as a function of sound pressure levels (speaking at higher intensity or singing seems to increase respiratory particle generation in the larynx)^24,33^.

### Emission rates

Table 2 shows the respiratory particle emission rates for speaking and loudly speaking tests. The emission rates for speaking and loudly speaking for the entire investigated population were significantly different, with median values of 26 and 41 particles s^−1^, respectively. The emission rates span a wide range, with 5th–95th percentile ranges of 7.1–93 and 10–146 particles s^−1^ for speaking and loudly speaking, respectively. A wide range of respiratory particle emissions was also reported in previous papers for adults and adolescents^22,25,33^. In Table 3, the existing data on respiratory particle concentrations and emission rates are summarized and compared with those obtained by this study. The emission rates for children for the present study were slightly lower or comparable to those obtained for children and adolescents by Fleischer et al.^23^ and Mürbe et al.^22^, respectively. Fleischer et al.^23^ also reported a higher emission rate for adults, and similar behavior was reported by Bagheri et al.^17^ in terms of particle concentrations. These two studies are of great relevance because they compare (adopting the same methodology) adults with children/adolescents. Other studies reported lower values for adults, but again, a proper comparison cannot really be performed due to the methodological differences amongst the studies. For example, Ahmed et al.^25^ found a median emission rate amongst adults of 9.1 particles s^−1^, but they performed a different phonation exercise, which may not be comparable with speaking activities.

**Figure 3:**
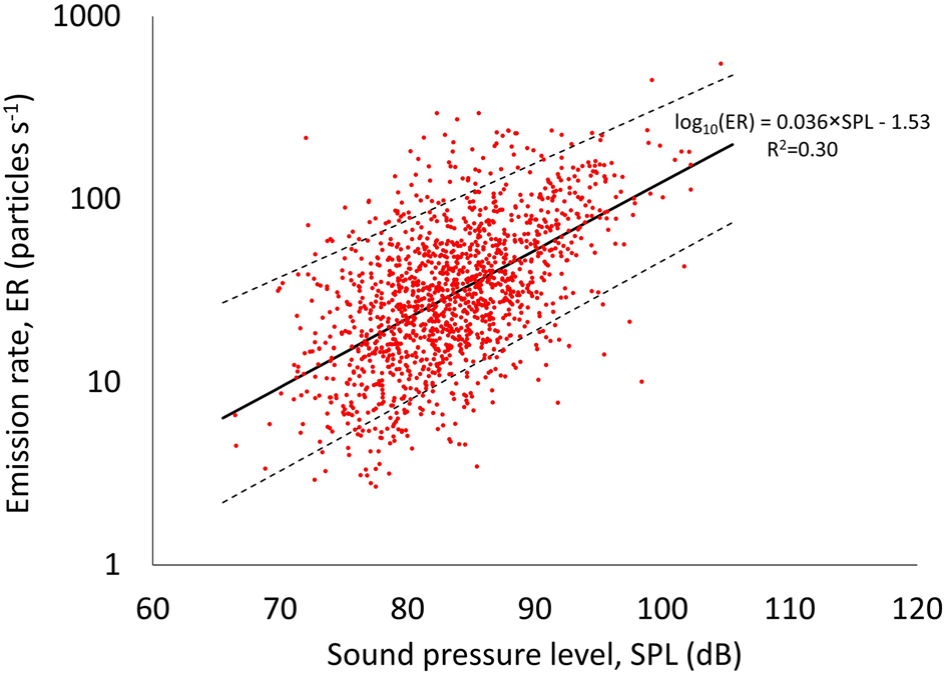
Respiratory particle emission rate (ER) as a function of the sound pressure level (SPL) including both speaking and loudly speaking. The black solid line represents the linear regression; the black dashed lines represent the 5th–95th percentile range.

Differences in emission rates between males and females are also reported in Table 2. In particular, males had significantly higher emissions than females both while speaking (median values of 28 and 23 particles s^−1^, respectively) and loudly speaking (51 and 33 particles s^−1^, respectively), consistent with the differences in particle concentrations (Table 2). In contrast, differences in emission rates due to the age of the child (Table 2) were not significant for either speaking or loudly speaking activities, even for age groups with different particle concentrations (e.g., 6-year-olds vs 12-year-olds). This result is likely due to the balancing of two opposite effects: the slight increase in the flow rate as a function of age (older children recorded statistically higher flow rates than younger children, Table 2) and the slight reduction in respiratory particle concentration with age (older children recorded statistically lower particle concentrations than younger children, Table 2). These emission characteristics clearly highlight an effect of the vocal loudness on the concentrations and emission rates. To quantify the vocal loudness effect, respiratory particle emission rates (ERs) are plotted against the sound pressure levels in Fig. 3. Here, the results for the entire population and both the speaking activities are plotted together. Figure 3 clearly shows an increase in the emission rates with the vocal loudness, which was also reported in previous studies^17,22,33^. In particular, an increase of 0.036 units of log_10_(ER) (R^2^ = 0.30) was found for a unit increase of the sound pressure level. Such an increase was slightly lower than that detected by Mürbe et al.^22^ for adolescents and by Archer et al.^27^ for a population mostly composed of adults (0.05 units of log_10_(ER)).

The findings of this study are of great significance as they could be applied to existing models to provide (despite their limitations^7,45^) predictive estimates of the risk of infection in indoor environments and/or in close-proximity configurations^7,8,14,15^. Indeed, such models are strongly dependent on the viral emission of the infected subject which is, in turn, influenced by the viral load carried by the respiratory particles (that can be obtained from PCR tests) and by the number of emitted particles. Thus, our findings are an important input for an effective simulation of the viral emission of children. Nonetheless, some limitations of the present study need to be pointed out: first of all, the study was performed considering children reading in Italian, anyway emission rates could be different in case of children reading phonetically balanced word lists in a different language. This aspect needs to be addressed in future researches. A further aspect that should be further investigated is the identification of the words, frequencies, letters mostly affecting the emission rates; indeed, the way we treated the data just allowed calculating an average emission rate characteristics of the entire phonetically balanced word list.

## Data Availability

The datasets used and/or analysed during the current study available from the corresponding author on reasonable request.
